# Jejunojejunal intussusception of a sutured enterotomy site after takedown and primary repair of persistent enterocutaneous fistula: a case report

**DOI:** 10.1093/jscr/rjac399

**Published:** 2022-09-20

**Authors:** Derek Marlor, Sibat Noor, Justin Beck, Khaled M Taghlabi, Mazin Al-Kasspooles

**Affiliations:** Department of General Surgery, University of Kansas Medical Center, Kansas City, KS, USA; University of Kansas School of Medicine, Kansas City, KS, USA; Department of General Surgery, University of Kansas Medical Center, Kansas City, KS, USA; Department of General Surgery, University of Kansas Medical Center, Kansas City, KS, USA; Department of General Surgery, University of Kansas Medical Center, Kansas City, KS, USA

## Abstract

Enterocutaneous fistula (ECF) is a common complication of many abdominal surgeries. Although most ECF resolve spontaneously, there are many factors that can lead to persistence of the fistula. Management of persistent enterocutaneous fistula usually involves surgery with recurrence of fistula being the most common complication. Here we describe a case of 67-year-old female who presented with intussusception following repair of a persistent enterocutaneous. Given the rare finding of intussusception in adults, this case report presents an interesting complication.

## INTRODUCTION

Enterocutaneous fistulas (ECFs) are known complications of abdominal surgery and following removal of enterostomy tubes. Although most gastrostomy or jejunostomy tracts undergo spontaneous closure, up to 3.7–8% experience persistent ECF, which is defined as leakage for greater than 1 month [1–3]. Persistent enterocutaneous fistulas (pECFs) are more common in adults than it is in children [4]. Complications of pECF include local skin irritation, abscess formation, sepsis, fluid collection, malnutrition and electrolyte imbalance [5]. Management of persistent ECF has traditionally been surgical; however, alternative methods including plugs, fulguration and endoscopic closures have been attempted with some success [6]. Surgical takedown of ECF is generally well tolerated and can be done laparoscopic or open and may be repaired primarily or may be resected with small bowel anastomosis. Complications of ECF takedown include recurrence, missed injury and standard abdominal surgical complications. No case reports exist which describe intussusception as a potential complication following ECF take down and primary repair of enterotomy. To our knowledge, we present the first documented case of intussusception at the previous ECF enterotomy suture repair site following ECF takedown, and the third case of intussusception with a sutured enterotomy site as the lead point [7, 8].

## CASE REPORT

We present the case of a 67-year-old female patient who presented to the clinic for the evaluation of surgical management of a 4-mm ECF at a prior jejunostomy (J-tube) site. Her J-tube had been removed 3 years prior and she experienced local skin irritation which had been managed with topical creams. Given the persistent symptoms of ECF, she was consented and subsequently taken to the operating room for enterocutaneous fistula takedown.

A small upper midline incision was made. Dissection was carried down to the fascia. The abdomen was entered. A persistent fistula was noted at the site of the previous jejunostomy tube. This was taken down with sharp dissection. Once freed from the abdominal wall, the jejunum was inspected and found to have a small enterotomy. The edges of the defect were ‘cleaned’ by excision of any unwanted tissue and subsequently closed with a running full thickness 3-0 PDS suture, followed by interrupted 3-0 Silk sutures. The abdomen was irrigated with warm saline, and the fascia and skin were closed in the standard fashion.

**Figure 1:**
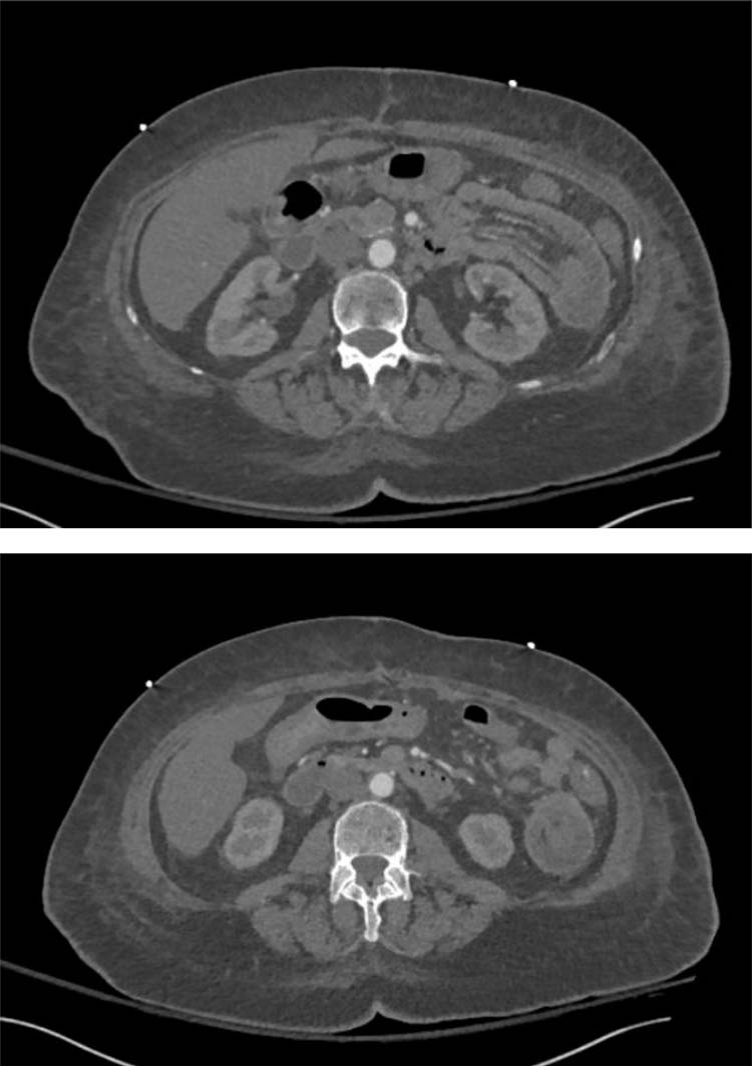
Intussusception of the proximal small bowel on CT abdomen without contrast.

Her initial hospital course was prolonged by poor oral intake. She was ultimately discharged on postoperative day (POD) 7. On POD 8, the patient was readmitted with nausea, emesis, failure to thrive, reported syncopal episode. Laboratory studies revealed diabetic ketoacidosis (DKA). She was transferred to the surgical intensive care unit (SICU) for management of suspected DKA. On hospital Day 2, a computed tomography (CT) scan of the chest, abdomen and pelvis was ordered due to leukocytosis and hemodynamic instability requiring vasopressor use and worsening abdominal pain. Contrast was not used due to an institutional shortage of IV contrast. The scan revealed atypical pneumonia and intussusception of the proximal small bowel (Fig. 1). Lactate at that time was 3.0 mM/l. Given the concern for possible ischemic bowel and recent surgical intervention, the patient was taken back to the operating room for exploration.

Using the prior midline incision, the abdomen was reopened. Approximately 10 cm from the ligament of Treitz at the site of the primary repaired fistula take down site, a jejunojenjunal intussusception was noted. The intussusception was reduced by applying distal pressure The bowel was noted to be hyperemic, but still viable. Given the concern for future recurrent intussusceptions with a proven lead point, a 15-cm small bowel resection was performed including the area of lead point and hyperemic bowel distally. Blue-loaded gastrointestinal anastomosis (GIA) stapler were used to create a side-to-side small bowel anastomosis. All staple lines were oversewn with interrupted silk sutures. The abdomen was closed in the standard fashion.

## DISCUSSION

Intussusception, or telescoping of the bowel, most often occurs in infants and children with typical presentation of abdominal pain, bloody stool and abdominal mass [9]. Adult intussusception, a rare finding accounts for only 5% of all intussusception cases, with > 90% of intussusception in adults due to secondary causes [10]. Tumors are the most common cause of intussusception in adults, and accounts for 63–77% of cases [11]. Other risk factors include anatomical changes, post-surgical adhesions, fibroids/polyps, inflammatory bowel disease gastrostomy tube and jejunostomy tube [12].

Intussusception can present in other iatrogenic settings such as a J-tube intussusception or following colonoscopy [13, 14]. However, unlike pathological intussusception, transient intussusception rarely requires surgical intervention and is often asymptomatic. Postoperatively, there are reports of intussusception after laparoscopic jejunorrhaphy for a jejunal perforation secondary to blunt abdominal trauma and ileo-ileal intussusception of a sutured enterotomy site [7, 8]. This perhaps occurs from ‘stickiness’ due to inflammation and/or scar formation at the repair site acting as a lead point for intussusception [15].

Our patient presented with hemodynamic instability and abdominal pain in the presence of DKA which prompted an emergent CT scan showing signs of atypical pneumonia and intussusception. She was taken to the operating room emergently due to her rapid clinical deterioration. Non-operative management of intussusception is often not recommended due to the known risks of a perforation from an obstruction, bowel ischemia and/or anastomotic complications. We recommend that providers keep intussusception on their differential and when identified to be at the site of bowel repair on imaging, emergent operative intervention should be undertaken.

## CONCLUSION

To our knowledge, there have been a limited number of documented reports of intussusception occurring postoperatively after abdominal surgery. To our knowledge, there has never been a documented report of an intussusception after a recent ECF repair; this potentially life-threatening complication should be considered when exploring the cause of an acute abdomen in these patients.
